# Prevalence of Gastrointestinal Parasites in Dogs and Potential Implications to Human Health in Accra, Ghana

**DOI:** 10.1155/japr/3973074

**Published:** 2025-05-30

**Authors:** Isaac Anim-Baidoo, Allotey Celia Naa Adorkor, Sherry A. M. Johnson, Thomas Koge Tingan, Akua Obeng Forson, Enid Owusu, Michael Olu-Taiwo, Eric Sampene-Donkor

**Affiliations:** ^1^Department of Medical Laboratory Sciences, School of Biomedical and Allied Health Sciences, University of Ghana, Accra, Ghana; ^2^School of Veterinary Medicine, University of Ghana, Accra, Ghana; ^3^Department of Medical Microbiology, University of Ghana Medical School, Accra, Ghana

**Keywords:** Accra, Ghana, community, dogs, gastrointestinal parasites, veterinary, zoonoses

## Abstract

Dogs provide security and companionship and enhance the psychological well-being of humans but also carry potential zoonotic pathogens posing a public health risk. This study was conducted to detect gastrointestinal parasites in dogs and determine their potential risk to human health. In all, 288 dog fecal samples were collected per rectum from a veterinary and community in Accra, Ghana. The samples were examined by flotation and sedimentation techniques and parasites identified using standard morphological criteria. Questionnaires were administered to dog owners for information on knowledge of zoonosis and pet management practices. The prevalence of gastrointestinal parasites in the dogs was 38.2% (110 infected out of the total 288 dogs from both veterinary and the community). The infection rate in community dogs was 70.8% (85 infected out of 120 dogs) and 14.9% (25 infected out of 168 dogs) in veterinary dogs. At the veterinary, three different parasites, namely, hookworm (11.3%), *Toxocara* spp. (2.4%), and taeniids (1.2%), were detected, while nine different species, including *Ancylostoma caninum* and *Toxocara* spp., were observed in community dogs. The study revealed a significantly higher prevalence of parasites in female dogs (41.4%) than in males (35.6%) (*p* < 0.001). Young dogs (1–40 weeks) recorded significantly lower prevalence (*p* < 0.05) than older ones. The highest prevalence among the dog species at the veterinary occurred in coonhound dark tan (50%) and pit bull (50%). There was a significant association between infection status and dog owners in the community who did not visit veterinary (*p* = 0.001) and veterinary dogs not dewormed (*p* = 0.003). No significant association existed between dog owners' knowledge of zoonosis and infection (*p* > 0.05). The odds showed that dogs of owners who did not visit veterinary and local domestic dogs were more likely to be parasitized. Our findings suggest a potential risk of dog parasites to human health and highlight the need to control the movement of dogs to public places and encourage dog owners to visit veterinary, as well as regularly deworm the dogs.

## 1. Introduction

Dogs have a close association with humans, providing security, a source of protein, and companionship and enhancing the psychological well-being of humans [1–3]. However, there are also potential health hazards associated with the ownership of a dog as a pet [3]. Apart from the risk of having bites, allergies, and scratches, dogs are also carriers of zoonotic pathogens [4–6]. The most common helminth parasites that infect dogs include *Toxocara canis*, hookworm, *Diphyllobothrium latum*, and *Echinococcus granulosus* [7–10], and the common protozoans among the parasites are *Giardia lamblia*, *Babesia* spp., *Cryptosporidium* spp., *Toxoplasma gondii*, and *Cystoisospora* spp. [11–14]. According to Lopes et al. [14], *T. gondii* is an important zoonotic parasite, although reports of epidemiological surveys and clinical cases of toxoplasmosis in humans and animals in the sub-Saharan countries of Africa are limited. They asserted that the potentially *T. gondii*-contaminated environment in Luanda may be responsible for the prevalence of *T. gondii* infection detected in dogs in their study [14]. Clinically, *Toxocara canis* can cause diarrhea, poor growth, and death if present in large numbers in dogs; and hookworm is known to be one of the most pathogenic helminths in dogs [7, 15]. In humans, the parasites hookworm and *Toxocara* spp. are responsible for the occurrence of cutaneous larva migrans (CLM) and visceral larva migrans (VLM), respectively [16, 17]. CLM consists of serpiginous linear papular lesions with an inflammatory aspect [18]. In VLM, the clinical findings include hepatomegaly, eosinophilia, and multiple oval lesions in the liver and lungs [19]. Dogs also serve as definitive hosts to echinococcosis, a disease of major public health concern [20, 21]. Research has shown that at least 36 significant zoonotic diseases are acquired from dogs worldwide [22]. There are reports that wind, rain, arthropods, human, and vehicular traffic can promote the spread of infective stages of parasites present in dog feces to human food and water sources [23–25].

The prevalence of gastrointestinal infections in dogs and their implication to public health have been reported worldwide [2, 9, 11, 12], and it is now clear that the uncontrolled spread of zoonotic infections from dogs to their owners is on the rise. In Ghana, information on the epidemiology of zoonotic parasites in dogs remains scanty. Almost a decade ago, the presence of zoonotic helminths in dogs was reported in Accra, Ghana, with a high prevalence of 62.6% [26] and an overall prevalence of 52.6% at Mampong, Ashanti region [27]. Recently, the involvement of other domestic animals in the possible transmission of zoonotic parasites has also been reported in the country. These include reports on the high prevalence of 32.2% toxocariasis in dogs, cats, and rodents [28] and 83.3% gastrointestinal parasites in cats [29]. Certainly, an update on these past reports through current research is long overdue.

Presently in the city of Accra, Ghana, there has been an increasing interest in keeping dogs due to their benefits as pets and for security reasons [1, 3]. This is evident in the frequent observation of the number of dog owners seen on the street with their dogs daily in many communities. Unfortunately, these dogs deposit their excreta freely during the walk to contaminate the environment [7], which remains on the street till they dry up. This appears to present a public health risk to people living in such communities, which could also possibly lead to a rapid spread of zoonotic parasitic infections [4–6, 9, 11, 12]. In addition, the population of unrestrained dogs, such as stray and farm dogs wandering freely in communities and who often defecate close to human habitations, has increased. Although the current situation demands a comprehensive study to ascertain the public health risk due to this occurrence, it has received little attention from researchers. There is thus only scanty information on the prevalence of gastrointestinal parasites in dogs and the potential risk of zoonotic transmission to human populations in the country.

To determine the risk of zoonotic transmission of parasites to dog owners and other animals in Accra, Ghana, we investigated the presence of gastrointestinal parasites in stool samples collected from dogs in communities and a veterinary clinic. Our study also provides current epidemiological data on dog owners' knowledge on zoonosis and pet management practices which may be relevant in the planning of effective control measures of zoonotic infections in animal and public health in the country.

## 2. Materials and Methods

### 2.1. Study Area

The study was conducted in Agbogba, a suburb of Accra located in the Ga East Municipal of Greater Accra region, Ghana. It has geographical coordinates of latitude 5°41⁣′0⁣^″^ north, longitude 0°12⁣′0⁣^″^ west and covers an area of 6.6 km^2^ with an elevation of 53 m above sea level. Based on the Ghana 2021 population and housing census [30], Agbogba has an estimated total population of 23,403, of whom 11,469 are males and 11,934 are females. The Small Animal Clinic (SAC) of the University of Ghana Veterinary School which was partly used for the study is situated close to Agbogba. Apart from training veterinary doctors, the facility offers veterinary services to the people in the area. It is equipped with a laboratory to examine clinical specimens collected from dogs and other animals brought to the clinic. On average, 15–30 dogs are brought to the clinic daily by their owners for medical care.

### 2.2. Fecal Sample Collection

A cross-sectional study which involved the collection of 288 dog fecal samples from the veterinary clinic and the community was conducted between March 2021 and October 2022. In the community, fresh samples were randomly collected from households by owners from the ground in the morning immediately after voiding by dogs and individually labeled in sterile, leakproof plastic containers. These samples were transported to the laboratory for immediate examination, while samples to be preserved were placed in 10% formalin. The age, gender, history of antihelminthic usage, and general information on dog management practices were recorded for each dog.

At the veterinary, fecal samples from the rectum of dogs were collected using swabs, containers, and zip lock covers and transported immediately to the parasitology laboratory of the facility for examination.

### 2.3. Parasitological Procedures

The fecal samples were examined macroscopically for adult nematodes and tapeworm proglottids. Each sample was subjected for examination by centrifugal fecal floatation technique using various solutions (saturated salt solution, zinc sulfate, and Sheather's solution) and formol ether sedimentation technique [31]. Briefly, three portions were obtained for each sample, and each portion was treated with one flotation solution. Eventually, each fecal sample was treated with all three samples to ensure uniformity in the sample treatment. Staining with iodine solution facilitated protozoan and cyst identification, and the modified Ziehl Neelsen (MZN) staining technique was used to detect oocysts in the feces [31, 32]. Parasites were identified using standard morphological criteria [33].

### 2.4. Assessment of Pet Management Practices and Awareness of Zoonotic Diseases

Questionnaires were administered to dog owners who consented to be interviewed. The questionnaires captured information on knowledge of zoonosis and pet management practices including deworming frequency, kennel cleaning frequency, and veterinary care.

### 2.5. Data Analysis

Raw data was entered into a Microsoft Excel spreadsheet, and descriptive statistics were used to summarize the data. The prevalence was calculated for all data as the number of infected individuals divided by the number of individuals examined and multiplied by 100 to express it as a percentage. Chi-square was used to assess the association of risk factors with the prevalence of parasites. The odds ratio (OR) with a 95% confidence interval (CI) was performed as a measure of association. All statistical analyses were performed using SPSS Version 16.0 (IBM, United States). *p* values less than 0.05 were considered statistically significant for all tests.

### 2.6. Ethical Approval and Consent of Owners

Ethical approval with identification number SBAHS/AA/MLAB/10730841/2021-2022 was obtained from the Ethical and Protocol Review Committee of the School of Biomedical and Allied Health Sciences (SBAHS), College of Health Sciences, University of Ghana. Also, verbal consent of the dog owners was obtained before the questionnaires were administered and dog fecal samples were collected.

## 3. Results

### 3.1. General Analysis

The study population, which comprised dogs sampled in the community (*n* = 120) and at the veterinary (*n* = 168), was made up of 56% (*n* = 160) males and 44% (*n* = 128) females. The overall prevalence of gastrointestinal parasites in the 288 studied dogs was 38.2% (110 infected out of the total 288 dogs from both veterinary and the community).

### 3.2. Distribution by Sample Groups (Veterinary and the Community)

The prevalence of the infection in community dogs was 70.8% (85 infected out of 120 dogs) and in veterinary dogs was 14.9% (25 infected out of 168 dogs). At the veterinary, three different parasites, namely, *Ancylostoma caninum* (19/168, 11.3%) *Toxocara* spp. (4/168, 2.4%), and taeniids (2/168, 1.2%) were detected in the dogs (Table 1). Comparatively, more gastrointestinal parasites were observed in the community dogs than in the veterinary dogs, and these were *Cryptosporidium* spp., *Ancylostoma caninum*, *Trichuris* spp., *Dipylidium caninum*, *Giardia* spp., *Cystoisospora* spp., *Fasciola* spp., and *Toxocara* spp., together with a few mixed infections of *Ancylostoma caninum + Toxocara* spp. and *Trichuris* spp. + *Ancylostoma caninum* (1/120, 0.8% each) (Table 1). Of these parasites, *Toxocara* spp. (28.3%) was the most common, followed by *Ancylostoma caninum* (19.1%). At the veterinary, regarding the intensity of infection (eggs excreted) of the various parasites, 2830 parasite eggs per gram were observed for *Toxocara* spp., 180/g for *Ancylostoma caninum*, and 1510/g for taeniids. In the community dogs, the intensities were 1205/g for *Cryptosporidium* spp., 1100/g for *Ancylostoma caninum*, 930/g for *Trichuris* spp., 1210/g for *Dipylidium caninum*, 1430/g for *Giardia* spp., 190/g for *Cystoisospora* spp., 120/g for *Fasciola* spp., and 2610/g for *Toxocara* spp. For almost all the parasites, the intensity of infection (eggs excreted) was higher (more than the averages described) in adult dogs from both community and veterinary compared to younger ones, with the exception of *Ancylostoma caninum.*

The study revealed an overall significantly higher prevalence rate of parasites in female dogs (41.4%) than in males (35.6%) (*p* < 0.001) (Table 2). In terms of age, dogs of 61–80 weeks old recorded the highest prevalence in both community and veterinary dogs, though not significantly higher than in other age groups (*p* > 0.05) (Table 2). While all the age groups of dogs studied in the community had various infection rates of gastrointestinal parasites, the age groups 41–60, 81–100, and above 100 weeks of dogs studied at the veterinary showed no infection. In general, young dogs of age 1–20 and 21–40 weeks recorded significantly lower infection rates (*p* < 0.001 and *p* < 0.040), respectively.

Dogs examined at the veterinary comprised different breeds, and while some of the breeds had no gastrointestinal parasite infections, others recorded varying prevalence rates (Table 3). The highest prevalence occurred in the coonhound dark tan (50%) and the pit bull (50%) followed by local dogs (37.1%), Malinois (33.3%), Scandal (25%), Caucasian shepherd (22.2%), Doberman (16.7%), husky (14.3%), and German shepherd (13.3%), with the lowest prevalence in poodle dogs (5.4%) (Table 3). Dogs examined in the community, on the other hand, were primarily local dogs.

### 3.3. Risk Analysis

The results of the questionnaires revealed a significant association between infection status and dog owners in the community who did not visit the veterinary (*p* = 0.001) (Table 4). Similarly, among the veterinary dogs, there was a significant association between infection status and dogs which were never dewormed (*p* = 0.003) (Table 5). No statistically significant association was observed between frequency of deworming in the community dogs, how regular kennel is cleaned, whether dog is domestic or semidomestic, dog owners' knowledge of zoonosis, feeding mode, and type of feed (Tables 4 and 5). The OR of the variables associated with the risk of gastrointestinal parasitism showed that dogs which did not visit veterinary (Table 4) and those that were local domestic dogs (Table 5) were all more likely to be parasitized. Also, how dog's feeding was done was also a factor that could contribute to dogs harboring intestinal parasites (Table 5).

## 4. Discussion

The study recorded an overall parasite prevalence of 38.2% among the 288 dogs sampled. This is comparatively lower than 49.9% [34] and 62.6% [26] previously reported in Accra and the Greater Accra region, respectively. It was also lower than 52.6% reported by Amissah-Reynolds et al. [27] in Mampong, Ashanti, also in Ghana. These differences in prevalence could be due to differences in research design as well as varying environmental conditions. The study in Accra involved carcass samples brought to the veterinary for autopsy, while that of Greater Accra, though from veterinary clinics and communities, involved a larger geographical area. The study from Mampong focused on specific areas, including those with marked poor environmental conditions. The prevalence is, however, in agreement with studies conducted in Poland, where an overall prevalence of 37.7% [35], in Alexandria, Egypt, of 40% among house dogs [2], 41.2% in Central Germany [11], and 41.7% from rural areas of the Czech Republic [36] were all reported. There are also lower prevalence rates reported from different parts of the world, including 21.5% in the northwest area of Mexico [9] and 5.87% in urban Qinghai Province, China [13]. In Mexico, the geographic location, where temperatures are high, contributed to the low prevalence. The authors explained that the high climatic temperatures suppress the development of parasite eggs, resulting in a low rate of viability, making it difficult for parasites to develop. In China, the report indicated that an effective monthly deworming program by the Chinese government was the reason for the low prevalence. In Ghana, exotic breeds of dogs that formed part of this study are better taken care of by their owners and may partly explain the comparatively lower prevalence observed, especially among dogs examined at the veterinary.

According to Anteson and Cockish [34], *Ancylostoma caninum* and *Toxocara* are the two intestinal parasites commonly diagnosed in dogs in Ghana. In recent studies in Ghana, *Toxocara* was reported in rodents [28] and cats [29]. In the present study, *Ancylostoma caninum* was identified as the most prevalent parasite in dogs examined at the veterinary clinic, followed by *Toxocara* spp. with prevalence of 11.3% and 2.4%, respectively. In the community dogs, *Toxocara* spp. (28.3%) was most prevalent, followed by *Ancylostoma caninum* (19.1%). Similar reports have been provided of these two parasites in other studies in Ghana [27] and other parts of the world [2, 7, 12, 13, 26] emphasizing the relevance of their zoonotic potential and associated risk of keeping dogs as pets.

It has been suggested that temperatures within the tropical areas are conducive for the development of parasites and could be part of the reasons for their high prevalence observed in many studies. In addition, most canine pets in Ghana are allowed household and sometimes neighborhood freedom, a practice that has been described by Anteson and Cockish [34] as a contributing factor to the spread of parasites in the environment. Stool deposition by these pets will make it readily possible for infections to be acquired and reacquired by them. The authors explained that under such circumstances, the occurrence and reoccurrence of the intestinal nematodes in a high percentage of dogs can be expected.

*Toxocara* spp. are soil-transmitted zoonotic helminths with their eggs being resistant to environmental factors and are viable for long periods in soil [37]. This could account for higher prevalence in community dogs compared to veterinary dogs since community dogs are mostly mobile and may easily come into contact with contaminated soil. Amissah-Reynolds et al. [37] reported *Toxocara* canis as the second most prevalent parasite in their study, which looked at zoonotic parasites from dogs in different agroecological zones in Ghana. Undoubtedly, this parasite is among those with a high level of endemicity in many areas [2, 7, 12, 13, 26, 27, 29, 37] including areas in the present study.

Similar to this study, Amissah-Reynolds et al. [37] also reported hookworm and *Fasciola* spp. among the zoonotic parasites from dogs in Ghana. The presence of these parasites in our environments, as demonstrated in our study, has a significant implication on public health which must not be ignored by public health authorities.

Although *Toxocara* infection is often subclinical or self-limiting, in many cases it may lead to a wide range of clinical presentations that depend on the degree of involvement of host tissue by the larvae [19]. Most important sites are the liver, lungs, heart, and CNS. Fever, hepatomegaly, liver necrosis, splenomegaly, eosinophilia, and myocarditis are the important clinical manifestations [16, 19]. *Ancylostoma caninum* infection causes CLM with clinical features varying from nonspecific dermatitis to typical creeping eruption [18]. An erythematous itchy papule is observed as the first lesion followed by about 2–3 mm thick, raised pink or flesh-colored swollen lesion. Later on, there is the formation of linear, serpentine (serpiginous), or bizarre tracks. In Manaus, capital of Amazonas State, North Brazil, it was reported that CLM was a disease of the poorest of the poor and a public health threat in the area but could be eliminated through improvement in living conditions [17].

Other GI parasites of public health concern like *Cryptosporidium* sp., *Giardia duodenalis*, taeniids, and *Dipylidium caninum* that were detected in the present study have been reported in many studies worldwide [2, 4, 5, 9, 20, 38, 39]. *E. granulosus*, which is considered zoonotic, is a parasitic pathogen that shares the morphological characteristics of the metacestode of the Taeniidae family [40]. Echinococcosis is a growing concern, possibly due to the worldwide distribution of *E. granulosus*, the difficulty of diagnosis and control, and the high cost and complexity of treatment [40]. Therefore, observation of taeniids in this study is of great concern.

Dogs are coprophagic and will eat not only dog feces but feces of many other animals as well. This may serve as a possible route of infection in the dogs as cysts of *Amoeba* and *Cryptosporidium* may be ingested, even though these infections could be acquired naturally. The ova/cyst of these parasites deposited in the environment could contaminate food or water and spread under poor hygienic conditions to affect human and animal health.

In general, the prevalence of gastrointestinal parasites was lower in the veterinary dogs, which could be attributed to the fact that they were restrained in their movement and dog owners provided better care, including regular deworming and visits to veterinary clinics than the community dogs. Similar findings were reported in Canada [41] and Havana, Cuba, where the prevalence of intestinal parasites was higher in stray dogs than in household dogs [42] because household dogs were given better attention by dog owners. Ahmed et al. [2] reported a lower prevalence of parasites in police dogs in Egypt than in house dogs and explained that the risk of infection was minimized in police dogs as a result of hygienic practices, regular deworming, and high-quality feeding.

Regarding breed, several exotic breeds such as Boerboel, Alsatian, dark tan mastiff, Maltese, Bernese mountain, Rottweiler, bulldog, and mongrel examined in this study all had no intestinal parasite infection compared with the local dogs, which agrees with observations made in Nigeria by Obioma et al. [7] who emphasized that exotic breeds are generally not stray dogs and are cared for by their owners. In Ghana, such exotic dogs are owned by individuals who can afford the cost of recommended feeding of dogs, protection, and general care of their dogs.

In this study, there was a significant effect of sex on the prevalence of intestinal parasites. Female dogs were significantly infected more than males, which agrees with many other studies [2, 43–45] but is contrary to findings by Ezema et al. [39] and Chidumayo [15] who reported more infections in males. Othman and Abuseir [46] who did not observe any significant difference in prevalence regarding sex explained that the dogs in their study in Palestine were exposed to similar conditions and infections and so the results were expected. The higher prevalence of intestinal parasites in the female dogs in the present study could be a result of a sedentary lifestyle during feeding and taking care of their puppies, while the males move around in search of mates. The high prevalence in females could also be attributed to hormonal influence on immunity, such as the periparturient relaxation of the immunity in pregnant and lactating females [33].

The prevalence of intestinal parasites in young dogs has been reported in many studies to be higher than in adult dogs [27, 41, 43, 47, 48], because of the parasite-specific immunity that develops with age, as a consequence of one or more exposures. However, the present study revealed a higher prevalence in adult dogs than in young ones, which contradicts these previous reports but is similar to others [38, 42, 45]. It could be due to the noncompliance of dog owners with scheduled deworming schemes, together with the exposure of dogs to contaminated environments. Also, there was a higher intensity of the infection (eggs excreted) in adult dogs than in younger dogs. This is worth noting, especially considering that *Toxocara canis* eggs excreted in older dogs were more than in younger ones. Among the dogs examined at the veterinary which were primarily exotic ones, however, some adult dogs recorded no infection. This could be a result of special care usually provided by dog owners to their exotic breed of dogs.

The investigation on the frequency of deworming, how regularly the kennel is cleaned, whether the dog is domestic or semidomestic, and dog owners' knowledge of zoonosis in this study as risk factors revealed no association with infection. However, keeping local breed domestic dogs and dog owners' attitudes of not visiting the veterinary were associated with infection risk. Generally, exotic dogs are given better care by owners than local ones, which are allowed to move freely in the community. In the process of wandering freely outside their homes unaccompanied, they defecate and eat anywhere in the environment and get infected and reinfected through contact with already contaminated soil or food. Dog owners who regularly visit the veterinary with their dogs obtain the medical treatment and guidance needed from veterinary doctors to keep their dogs healthy and free of intestinal parasites. It is necessary that veterinary care and public health education are increased in order to protect dogs and their owners. Dogs must be given restrictions from going to public places so that the rate of contamination of the environment by dog fecal matter can be reduced, which will consequently reduce the risk of human infection by dog parasites. Although it is now common to see dog owners in the company of their dogs on the streets, there could be less risk of intestinal parasite infection associated with exotic dog breeds than local ones, which in most cases move around unaccompanied by owners. The public health implications of dogs moving unaccompanied in public places cannot be ignored. The risk of zoonotic transmission of parasites could be reduced if owners are educated to collect fecal samples dropped by their own pets in public areas and to regularly check the parasitic status of their dogs.

## 5. Conclusion

In conclusion, our results revealed that dogs at the community and veterinary were both infected with zoonotic species of gastrointestinal parasites. The most prevalent parasites identified were *Ancylostoma caninum* and *Toxocara* spp., which can be a serious threat to public health. Local breeds of dogs together with dogs whose owners do not regularly visit the veterinary were at a higher risk of infection. These observations highlight the need to control the movement of dogs to public places as well as encourage dog owners to regularly visit the veterinary where they can receive education on healthy practices in keeping dogs. Moreover, deworming dogs should be considered an important practice and done regularly.

### 5.1. Limitations of the Study

The parasites observed under the microscope were not measured in this study. It is recommended that parasites be measured in future studies since this would help with species determination, for example, *Trichuris vulpis* eggs measure 70–85 × 35–40 *μ*m, which clearly distinguishes them from other species of the genus *Trichuris*.

## Figures and Tables

**Table 1:** Frequency of gastrointestinal parasites in fecal samples of dogs from the veterinary clinic of the University of Ghana Veterinary School and the Agbogba community, between March 2021 and October 2022 (*n* = 288).

	**Name of parasite**	**Positives**	**Frequency (%)**
Veterinary dogs (*n* = 168)	*Ancylostoma caninum*	19	11.3
	*Toxocara* spp.	4	2.4
	Taeniids	2	1.2

Community dogs (*n* = 120)	*Cryptosporidium* spp.	1	0.8
	*Ancylostoma caninum*	23	19.1
	*Trichuris* spp.	2	1.7
	*Dipylidium caninum*	5	4.2
	*Giardia* spp.	8	6.7
	*Cystoisospora* spp.	7	5.8
	*Fasciola* spp.	3	2.5
	*Toxocara* spp.	34	28.3
	Mixed infections		
	*Ancylostoma caninum + Toxocara* spp.	1	0.8
	*Trichuris* spp. + *Ancylostoma caninum*	1	0.8

**Table 2:** Age group and sex-related prevalence of gastrointestinal parasites in dogs from community (*n* = 120) and veterinary (*n* = 168).

Veterinary dogs	Age (weeks)	No. examined	No. positive	Prevalence (%)	*p* value
	1–20	108	19	17.59%	
	21–40	34	5	14.71%	**<0.001**⁣^∗^
	41–60	8	0	0	
	61–80	3	1	33.33%	
	81–100	5	0	0	
	> 100	10	0	0	
	Sex				
	Male	104	16	15.38	
	Female	64	9	14.06	**< 0.001**⁣^∗^

Community dogs	Age (weeks)				
	1–20	22	16	72.73	**0.026**⁣^∗^
	21–40	24	17	52.50	0.411
	41–60	15	11	73.33	0.0511
	61–80	10	8	80.0	0.521
	81–100	12	7	58.33	0.567
	> 100	37	26	70.27	**0.012**⁣^∗^
	Sex				
	Male	56	41	73.21	
	Female	64	44	68.75	**0.004**⁣^∗^

All dogs	Age (weeks)				
	1–20	128	35	27.34	**< 0.001**⁣^∗^
	21–40	58	22	37.93	**0.040**⁣^∗^
	41–60	23	11	47.83	0.417
	61–80	13	9	69.23	0.098
	81–100	17	7	41.18	0.237
	> 100	47	26	55.32	0.234
	Sex				
	Male	160	57	35.63	
	Female	128	53	41.41	**< 0.001**⁣^∗^

⁣^∗^*p* values less than 0.05 and were considered statistically significant.

**Table 3:** Dog breed and prevalence of gastrointestinal parasitic infection among dogs (*n* = 168) examined at the veterinary.

**Name of dog breed**	**No. examined**	**No. infected**	**% infected**
Boerboel	10	0	0
German shepherd	15	2	13.3%
Local	35	13	37.1%
Mixed breed	15	0	0
Husky	7	1	14.3%
Poodle	37	2	5.4%
Caucasian shepherd	9	2	22.2%
Alsatian	7	0	0
Dark tan mastiff	1	0	0
Maltese	4	0	0
Doberman	6	1	16.7%
Malinois	3	1	33.3%
Coonhound dark tan	2	1	50%
Bernese mountain	6	0	0
Scandal	4	1	25%
Pit bull	2	1	50%
Rottweiler	1	0	0
Bulldog	3	0	0
Mongrel	1	0	0

**Table 4:** Binary logistic regression results for the presence of gastrointestinal parasite infection in dogs from the community (*n* = 120).

**Variable**	**Categories**	**No. examined**	**No. positive**	**Prevalence (%)**	**Odds ratio** **95% CI**	**p** ** value**
Veterinary visitation	Yes	22	4	18.18	0.93, 0.28–3.105	0.454
	No	98	81	82.65	101.6, 50.1–206.1	**0.001**⁣^∗^

Frequency of deworming	Once every 3 months	15	7	46.67	1.48, 0.54–4.09	0.220
	Once every 6 months	36	17	47.22	1.01, 0.471–2.169	0.489
	Once a year	12	7	58.33	1.010, 0.3–3.47	0.493
	Never	57	54	94.74	1.144, 0.24–4.542	0.467

How regularly kennel is cleaned	Daily	85	56	65.88	0.99, 0.537–1.824	0.487
	Weekly	25	21	84.0	1.0, 0.303–3.305	0.500
	Monthly	3	2	66.67	1.0, 0.087–11.43	0.514
	None	7	6	85.71	1.01, 0.113–9.03	0.498

Type of dog	Domestic	107	76	71.03	1.0, 0.545–1.83	0.495
	Semidomestic	13	9	69.23	1.27, 0.395–4.134	0.341

Dog owners' awareness of zoonosis	Yes	32	23	71.88	0.992, 0.401–2.407	0.493
	No	88	62	70.45	1.012, 0.409–1.901	0.482

How feeding is done	In a bowl	82	58	70.73	0.998, 0.553–1.9	0.498
	On the floor	31	22	70.97	0.987, 0.48–2.4	0.488
	Both	7	5	71.43	1.014, 0.156–5.536	0.493

Type of feed	Dog feed	19	8	42.11	1.01, 0.375–2.738	0.489
	Raw meat and household leftover	101	77	76.24	0.877, 0.465–1.655	0.345

⁣^∗^*p* values less than 0.05 and were considered statistically significant.

**Table 5:** Binary logistic regression results for the presence of gastrointestinal parasite infection in dogs from the veterinary (*n* = 168).

**Variable**	**Categories**	**No. examined**	**No. positive**	**Prevalence (%)**	**Odds ratio** **95% CI**	**p** ** value**
Veterinary visitation	Regular	89	11	12.36	0.978, 0.408–2.342	0.480
	Not regular	79	14	17.72	0.965, 0.442–2.047	0.461

Frequency of deworming	Once every 3 months	26	2	7.69	2.054, 0.412–10.24	0.189
	Once every 6 months	61	7	11.48	0.964, 0.352–2.638	0.471
	Once a year	76	13	17.11	1.0, 0.454–2.221	0.495
	Never	5	3	60.0	0.625, 0.09–0.45	0.003⁣^∗^

How kennel is regularly cleaned	Daily	142	18	12.68	0.950, 0.455–2.073	0.449
	Weekly	26	7	26.92	0.997, 0.366–2.564	0.472
	Monthly	0	0	0		
	None	0	0	0		

Type of dog	Domestic	162	21	12.96	3.20, 1.721–5.972	0.001⁣^∗^
	Semidomestic	6	4	66.67	0.096, 0.06–0.571	0.005⁣^∗^

Dog owners' awareness of zoonosis	Yes	131	15	11.45	0.097, 0.432–2.203	0.500
	No	37	10	27.03	1.01, 0.453–2.35	0.488

How feeding is done	In a bowl	142	9	6.34	0.955, 0.332–2.77	0.465
	On the floor	10	4	40.0	0.444, 0.147–1.346	0.081
	Both	16	12	75.0	31.5, 14.94–66.37	0.567

Type of feed	Dog feed	138	6	4.35	5.54, 0.611–50.51	0.067
	Raw meat and household leftover	30	19	63.33	1.013, 0.432–2.360	0.487

⁣^∗^*p* values less than 0.05 and were considered statistically significant.

## Data Availability

The data that support the findings of this study are available from the corresponding author upon reasonable request.
